# Phytogeographical Analysis and Ecological Factors of the Distribution of *Orchidaceae* Taxa in the Western Carpathians (Local study)

**DOI:** 10.3390/plants10030588

**Published:** 2021-03-20

**Authors:** Lukáš Wittlinger, Lucia Petrikovičová

**Affiliations:** Department of Geography and Regional Development, Faculty of Natural Sciences, Constantine the Philosopher University in Nitra, 94974 Nitra, Slovakia; wittlingerl@gmail.com

**Keywords:** orchid Species, species surveys, endangered species, expansion area, abiotic environments, ecological amplitude

## Abstract

In the years 2018–2020, we carried out large-scale mapping in the Western Carpathians with a focus on determining the biodiversity of taxa of the family *Orchidaceae* using field biogeographical research. We evaluated the research using phytogeographic analysis with an emphasis on selected ecological environmental factors (substrate: ecological land unit value, soil reaction (pH), terrain: slope (°), flow and hydrogeological productivity (m^2^.s^−1^) and average annual amounts of global radiation (kWh.m^–2^). A total of 19 species were found in the area, of which the majority were *Cephalenthera longifolia*, *Cephalenthera damasonium* and *Anacamptis morio*. Rare findings included *Epipactis muelleri*, *Epipactis leptochila* and *Limodorum abortivum*. We determined the ecological demands of the abiotic environment of individual species by means of a functional analysis of communities. The research confirmed that most of the orchids that were studied occurred in acidified, calcified and basophil locations. From the location of the distribution of individual populations, it is clear that they are generally arranged compactly and occasionally scattered, which results in ecological and environmental diversity. During the research, we identified 129 localities with the occurrence of 19 species and subspecies of orchids. We identify the main factors that threaten them and propose specific measures to protect vulnerable populations.

## 1. Introduction

Human activity and its impact on the natural environment have a significant impact on biological systems. These activities have a particularly negative impact on wild fauna and flora, disrupting the links and relationships between these systems. In this article, we focus on taxa of the family Orchidaceae, which react very sensitively to changes in the environment. Many species are critically endangered. Anthropogenic interventions that cause their direct retreat can include uprooting in gardens and the use of phytotherapeutic effects, but indirect interventions are more significant, such as the intensification of agriculture, forestry, land drainage, soil compaction, etc. In such cases, the protection of biodiversity is an important part of our understanding. In the Slovak Republic, all species of wild orchids are protected except Neottia nidus-avis, and they are included in the Red List of Spore and Flowering Plants of Slovakia [1]. This fact is also emphasized by the Nature and Landscape Protection Act [2].

Slovak orchids are also protected by international conventions by which the Slovak Republic is bound, including the Convention on International Trade in Endangered Species of Plants and Animals, the Washington Convention, or CITES, and the Convention on the Conservation of European Wildlife and Habitats, or the Berne Convention [3]. In the European Union, the Berne Convention is Directive no. 92/43/EHS regarding the conservation of the habitats of wild fauna and flora.

Geophytic species of orchids are characterized by their occurrence in limestone substrate. Orchids occur in most mesotrophic habitats (in soil profiles moderately supplied with water). From the point of view of soil acidity, they belong within alkalophytes to neutrophytes (pH 6.8–7.2). Most of our native orchid species are tied to warm areas in lower positions [4]. Some authors [5] state that most orchids belong among calcific to basophilic species. We investigated whether these statements applied in general to the territory of the Western Carpathians. Despite the initial field surveys of the area, we agreed that the occurrence of orchids was concentrated mainly in part of the karst area (Tuhársky karst). Through further surveys in 2019 and 2020, we confirmed a higher number of localities with the occurrence of orchids just outside the areas that were built of limestone. It was mostly acidic plutonium (alkaline rocks). This was a reason to investigate the cause and our conclusions determined that orchids within ecological groups are represented by calcifytes to basifytes, respective alkalophytes of subsoil ground.

Research carried out in the Slovak Republic and other countries confirms that significant factors of distribution are the geological conditions of the area, which has a considerable impact on their occurrence and overall phytogeographical distribution of these species, as well as indicative values of populations, their communities and spatio-temporal changes in occurrence; these claims can be defined on the basis of the relationship to the environment and the influence of environmental factors. Similar research has been carried out in the Western Carpathians [6] and in Europe by several authors [7,8,9,10,11,12,13,14].

Within Europe, a wide range of research results have been available on the spread of orchids in the context of environmental factors. A study from Greece explains the spatial distribution of endangered orchids by land area and area of calcium substrates [15]. A study from long-term monitoring of the population of *Cypripedium calceolus (Orchidaceae)* in Italy identifies the intensification of forestry as a significant factor in population decline [16], which also appears to be a significant problem in the Western Carpathians. Botanical research in the British Isles [17] has shown that more open growth is benefiting from the increase in orchid species, as evidenced by our mappings, as meadow species have shown higher vitality through constant extensive mowing or the grazing of sheep and cattle. A study from the Russian Federation (Komijsko) states that the size of the orchid population is influenced by the climatic conditions of the current, as well as the previous growing season. The population is positively affected by the correlation of temperature and humidity [18]. In the territory of the Western Carpathians, orchids are hemisciophytes to sciophytes, which bind mainly to moderately warm habitats in lower positions. Orchid species in China are one of the most species-rich families and endangered groups of plants [19]. Most orchids are narrowly distributed in specific habitats due to their mycorrhizal specificity, specialization of pollinators and limited seed germination. In comparison with plants from other families, orchids are extremely susceptible to habitat disturbance, which has been significantly demonstrated in the area we studied. However, little is known about how orchids are distributed and how they are protected on a large scale. That is why we carried out biogeographical research using the method of large-scale mapping.

The aim of the research was to map the species diversity and abundance of orchids in the studied area of the Western Carpathian Mountains (geomorphological units Revúcka vrchovina Mountains, Stolické vrchy Mountains and Veporské vrchy Mountains). Within the distribution, we recorded localities with the occurrence of these species. We then defined the basic ecological factors of localities and statistically evaluated them and compared their impact on the spread of orchids. The study represented field research in the period 2018–2020. The area was mapped on the basis of cartographic outputs (geology, pedology, potential vegetation), and localities were selected which, based on ecological factors, represented possible habitats inhabited by orchids. In the second stage of the mapping, the whole area was examined in order to supplement the localities where these species were less likely to occur. Each identified locality received its own code, recording GPS coordinates; taxon; substrate: ecological value of the land unit; soil reaction (pH); terrain: slope, flow and hydrogeological productivity; and the average annual amount of global radiation. We obtained data from cartographic outputs and verified them in the field (rock verification, excavation of the soil profile, etc.).

Prior to the actual field research, we believed that most of the orchids would be concentrated in localities that were built of limestone substrate. The findings surprised us to a large extent, as most of the studied species preferred localities with acidic rocks. Another of the research questions was whether the important ecological factors of the environment included only the geological structure of the area, or whether other ecological factors of the area also had an impact and to what extent.

## 2. Materials and Methods

### 2.1. Study Area

**Figure 1:**
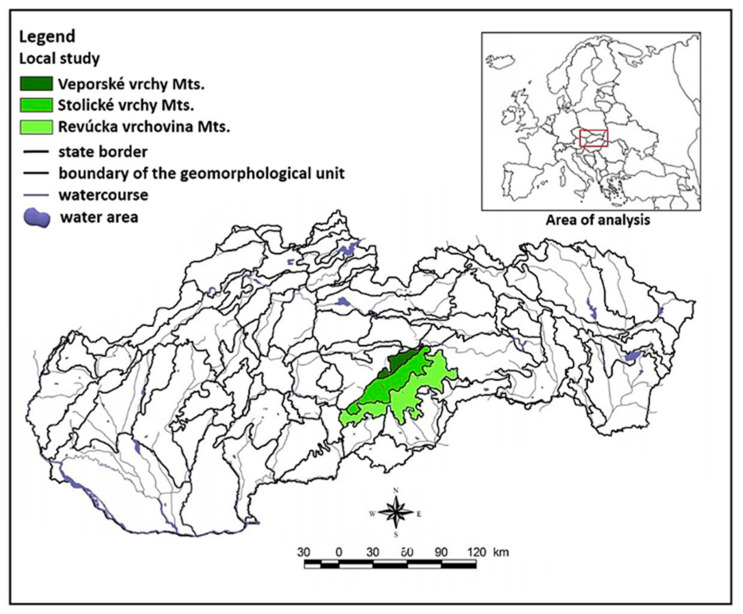
Map of the studied area.

The studied area of the Western Carpathian Mountains is located in the south of Central Slovakia (Central Europe) and defined by geographical coordinates between 48.42°–48.61° latitude and 19.76 –19.66° longitude at an altitude of 283 to 1110 m (Figure 1). The area is geographically part of the Alpine–Himalayan system, belongs to the Carpathian subsystem and is divided into the provinces of the Western Carpathians and the Slovak Ore Mountains. Furthermore, its area extends to the Revúcka vrchovina Mountains, Stolicke vrchy Mountains and Veporské vrchy Mountains. [20]. This research does not deal with the whole area of interest, but with a local study within the three mentioned units with an area of 414,881 km^2^.

### 2.2. Geographical Conditions

From the phytogeographical point of view, the studied area belongs to the crystalline-Mesolithic area of the oak and beech zones. The vegetation cover of this area is very differentiated thanks to the diverse petrographic background and richer shaped relief, which affects the vegetation, especially through soil conditions [21]. The complex geological structure is dominated by cuffs, gneisses, filites and granites. Lower Triassic quartzites, limestones and Neogene andesite tuffs also occur in some parts of the mountain range. The extremely varied relief has a predominantly highland character [22].

The area belongs to the Ipeľ and Slaná river basins. The climate in this area is continental. The climatic–geographical type represents a mountain climate [23]. The dominant soil type is Cambisol, which occurs in the area in the subtype Haplic Cambisol, accompanying Anthrosol and Leptosol, from the weathering of acidic to neutral rocks; than Planosol in the subtype Haplic Planosol (cultured, luvised and saturated move to acidic—from loess clays and slopes); Leptosols in the subtypes Rendzic Leptosols, Rendzic Lithic Leptosols and Lithic Leptosol carbonate (on limestone); places with shallow substrates of the terrae calcis type and Gelyic fluvisol and accompanying gleys from carbonate and non-carbonate alluvial sediments [24].

Vegetation is represented by a spectrum of different grasslands and to a greater extent, forests. This area is dominated by habitats such as floodplain and mountain floodplain forests *(Alnenion glutinoso-incanae* Oberd. 1953; *Salicion triandrae* Th. Műller et Gőrs 1958 p.p; *Salicion eleagni* Moor 1958), Carpathian oak-hornbeam forests *(Carici pilosae-Carpinenion betuli* J. et M. Michalko 1986), flowering beech forests *(Eu-Fagenion* Oberd 1957 pp maj), oak-cerium forests *(Quercetum petraeae-cerris* Soó 1957 s. l.) [25].

### 2.3. Landscape Ecology and Landscape Cover Analysis

The analysis of landscape cover is one of the basic sets of data on the current state of the landscape, taking into account the socio-economic activities of man. It is this data that can tell us how human activity and its spatio-temporal changes affect the biodiversity of the studied area. Under the Corine Land Cover program [26], we can analyze the current state of land cover and land use.

From the point of view of landscape ecology, it is important to create an analysis that will allow us to better understand the inter-relationships between human society and the surrounding natural environment, the structure of ecosystems, changes taking place in them, ecosystem development, interrelationships between organisms and plants and animals. Based on the analysis of landscape cover, we identified factors that significantly contributed to the degradation of native habitats as well as to specific populations of orchids.

We can study the changing identity of a territory in several possible ways. For our research, we have chosen the traditional approach, which understands the territory as a holistic system that changes its identity during historical development. It is a functional–spatial development and morphogenetic analysis of the changing use of land and spatial structure as well as housing construction and social change. From a geographical perspective, we attempt to explain the basic characteristics of the territory, which, during its development, has caused a continuous impact on the territory in a wider spatial context— suburbanization, regional transformations and their main factors, such as settlement changes, etc.

The predominant part of the studied area consists of continuous and discontinuous urban structures, in the vicinity of which industrial or commercial units are built.

**Figure 2:**
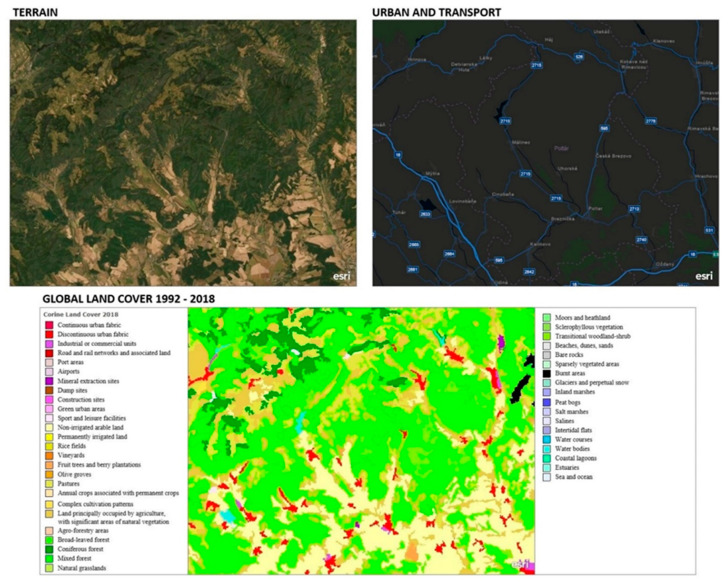
Map of the landscape cover of the studied area. [26].

The transport network includes road and rail transport and related land. The town of Lučenec has a dominant position in the studied area, which is situated at the crossroads of the main road and railway routes connecting Bratislava with Košice and Warsaw with Budapest. We are currently working on the construction of an expressway on the international route (Brno–Trenčín–Zvolen–Rimavská Sobota–Košice) (Figure 2).

From the facilities of the production and non-production spheres, there are localities in the area where rocks and minerals are currently being mined. One of the localities is Mýtna-Hrby, where the population of *Orchis purpurea* [27] is located as well as green urban areas and sports and recreational facilities.

Of the land types, the predominant part of the territory consists of non-irrigated arable land, permanently irrigated land, orchards, vineyards, permanent grasslands (pastures), fields with annual crops associated with permanent crops, land predominantly inhabited by agriculture with significant areas of natural vegetation, forestry areas, deciduous forests, coniferous forests, mixed forests, natural grasslands, transitional forests, watercourses and artificial reservoirs.

We analyzed the current state of land cover and land use by the Land Cover map layer implemented in ArcGIS by ESRI. The analysis confirmed the significant impact of human socio-economic activity, which had a high share in the loss of diversity of orchids as well as natural habitats in which the populations are located. Subsequently, we combined the analysis with the reconnaissance of the studied area and determined the main factors that threaten the populations.

Factors threatening the *Orchidaceae* population (own field research):

- anthropogenic - intensification of agriculture; forestry management; drainage; land use for technical construction; harvesting for gardening and medical purposes; mineral extraction; application of fertilizers; application of herbicide;, large-scale application of insecticides; soil compaction; mulching—covering of the soil surface
- zoogenic - damage done by large ungulates, small subterrestrial mammals and insects
- phytogenic - displacement by non-native (invasive) plant species, succession—plant association change
- pedogenic - soil erosion, high pH, nitrogen and ion content in soil.

### 2.4. Data Analyses

In the years 2018–2020, we carried out large-scale mapping and monitoring of orchid species (a total of 19 species were found in the study area). The results of monitoring can be used to prepare reports on the state of habitats and species of European importance for the European Commission and thus meet national legislative requirements and the requirements of EU directives. We performed the mapping through field biogeographic research at selected localities according to established methodology and frequency. Non-forest and forest habitats and botanical taxa of the *Orchidaceae* family were monitored. As part of the expansion of knowledge and requirements of the State Nature Conservancy of the Slovak Republic (hereafter SNC SR), we also mapped the species *Neottia nidus-avis*, which is currently not endangered or protected. The number of monitored localities where we recorded the occurrence of orchids was 129, while at 33 localities we monitored *Neottia nidusavis*.

We obtained basic data on the distribution of orchids from the Comprehensive Information System of SNC SR. Subsequently, we verified the data in the field. We recorded all the findings by photograph and GPS recording (WGS 84) [28]. We presented the scientific names of taxa in the sense of the work [1,29]. The next step was to obtain data on environmental factors for statistical evaluation. We drew geological information from the World Lithology Map [30]. We also worked with data from the World Soil Map, with respect to soil response [31]. We used knowledge about the slope of the relief and its orientation to the sides of the world from the Terrain: Slope Map application [32]. We drew the algebraic sum of fluxes of direct and scattered radiation falling on a horizontal surface, which formed the flow of global radiation and represented the basic component of the sun’s total radiant energy intake on the earth’s surface from the Global Radiation and Relative Sun Duration map [33]. The last part was data on hydrogeological conditions with the size of the coefficient of flow (transmissivity) [34]. We used Ellenberg Indicator Values [28] to characterize individual indicator values for individual factors. It was a phytogeographical and ecological analysis, and it was a procedure that evaluated the properties of the environment on the basis of phytoindication. The evaluation was based on knowledge of the habitat needs of individual plants with respect to their environmental requirements. In Europe, the most used are the so-called Ellenberg Indicator Values that enable statistical evaluation and more precise characterization of plant communities. This methodology was also addressed by the authors [5].

## 3. Results

### 3.1. Monitored Taxa

Mapped taxa and their legal protection within the Slovak Republic (§) endangerment in accordance with IUCN.

Least Concern (LC), Near Threatened (NT), and Vulnerable (VU) [1]:

1. *Cephalanthera damosonium* (Mill.) Druce, NT / §
2. *Cephalanthera longifolia* (L.) Fritsch, NT / §
3. *Cephalanthera rubra* (L.) Rich., NT / §
4. *Anacamptis morio* (L.) R. M. Bateman, NT / §
5. *Dactylorhiza majalis subsp. majalis* (Rchb.) P. F. Hunt et Summerh, NT / §
6. Dactylorhiza sambucina (L.) Soó, NT/ §
7. Orchis purpurea Huds., NT / §
8. Dactylorhiza fuchsii subsp. fuchsii (Druce) Soó, NT / §
9. Platanthera bifolia subsp. latiflora (L.) Rich., LC
10. *Platanthera chlorantha* (Custer) Rchb., NT / §
11. Epipactis helleborine subsp. helleborine (L.) Crantz, LC
12. *Epipactis atrorubens* (Hoffm.) Besser, LC / §
13. *Epipactis microphylla* (Ehrh.) Sw., LC / §
14. Epipactis muelleri Godfery, NT / §
15. Epipactis leptochila Godfery, VU / §
16. *Limodorum abortivum* (L.) Sw. NT / §
17. *Neottia nidus-avis* (L.) L. C. M. Richard
18. *Gymnadenia conopsea* (L.) R. Brown., LC / §
19. *Gymnadenia odoratissima* (L.) Rich., NT / §

Within the genera, the genera *Epipactis* (28%), *Cephalanthera* (17%) and *Dactylorhiza* (17%) had the largest representation. The lowest representation was of the genera Orchis and *Anacmaptis* (5%). From an ecosozological point of view, 84% of species in the territory of the Slovak Republic are protected by law. In addition, species are included in the NT category (67%) of the mapped species, 28% of species belong to the LC category and only one species falls into the VU category, which represents 5%.

**Figure 3:**
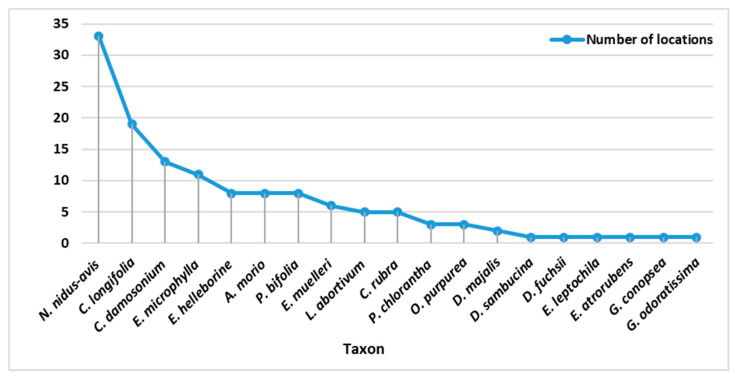
Taxa and number of detected localities.

**Figure 4:**
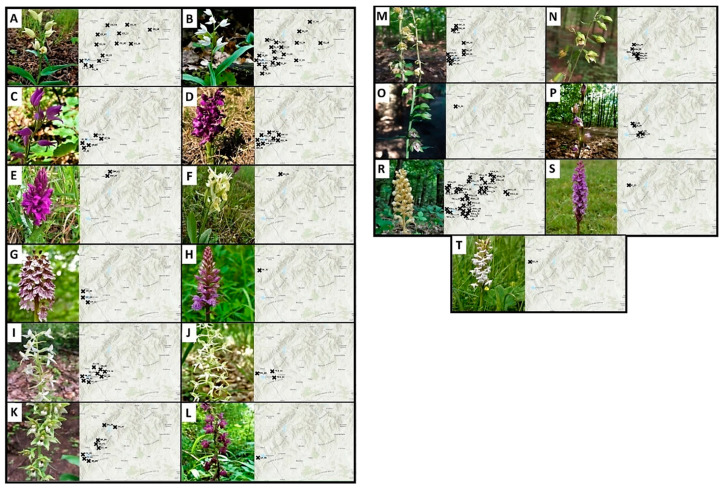
Distribution of orchids in the Western Carpathians: (**A**) Cephalanthera damosonium, (**B**) Cephalanthera longifolia, (**C**) Cephalanthera rubra, (**D**) Anacamptis morio, (**E**) Dactylorhiza majalis, (**F**) Dactylorhiza sambucina, (**G**) Orchis purpurea, (**H**) Dactylorhiza fuchsii, (**I**) Platanthera bifolia, (**J**) Platanthera chlorantha, (**K**) Epipactis helleborine, (**L**) Epipactis atrorubens, (**M**) Epipactis microphylla, (**N**) Epipactis muelleri, (**O**) Epipactis leptochila, (**P**) Limodorum abortivum, (**R**) Neottia nidus-avis, (**S**) Gymnadenia conopsea, (**T**) Gymnadenia odoratissima.

The number of species and represented localities was not balanced. Of the species studied, *Neottia nidus-avis* (25%), *Cephalanthera longofilia* (20%), *Cephalanthara damasonium* (10%) and *Epipactis microphylla* (8%) had the largest proportions. The least represented localities were *Platanthera chlorantha* (2%), *Epipactis leptochila* (1%), *Epipactis atrorubens* (1%), *Gymnadenia conopsea* (1%) and *Gymnadenia odoratissima* (1%) (Figure 3 and Figure 4).

### 3.2. Ecological Factors and Diversity of Orchids

Most of the studied orchids occur in acidophilic, calcific to basophilic habitats, i.e., they are acid-based (in the soil profile slightly to moderately supplied with water) [35,36,37,38]. In relation to soil acidity, it can be argued that most of the mapped species belong to acidophytes (pH 4.5–5.5) or mesophytes (pH 5.5–7.2). Some species with a wide ecological amplitude grow on limestone soils—calcifytes to basifytes. The studied orchids represent hemisciophytes to sciophytes, which are mainly bound to moderately warm habitats in lower positions.

Ref. [39] states that within the ecological group of species, most taxa of *Orchidaceae* belong to the group of calcific to basophilic species. This group represents species associated by their occurrence on soils with a high content of bases with a slightly acidic, neutral to alkaline reaction—especially calcium, but also magnesium and potassium. The focus is on soils formed from limestones and dolomites (Leptosols), but also other rocks rich in bases (melaphyrs, basalts, andesites, etc., or loess). The area we studied is built mainly by volcanic subsoil. The occurrence of orchids on such a subsoil is explained by [5] only by specific, long-term stable use. This method, of which we do not know much yet, seems to lead to lower interspecies competition, which has enabled the survival of populations of several species of *Orchidaceae*. These sites require constant care to achieve optimal natural conditions, such as the removal of overgrowth and invasive plant species (Table 1).

**Figure 5:**
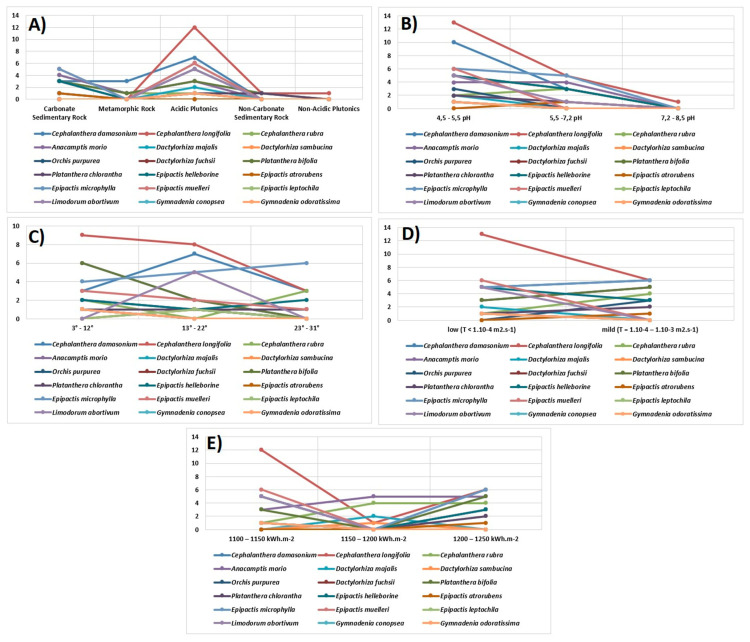
Graphical evaluation of ecological factors: (**A**) Substrate: ecological land unit value, (**B**) Soil reaction (pH), (**C**) Terrain: slope (°), (**D**) Flow and hydrogeological productivity (m^2^.s^−1^), (**E**) Average annual amounts of global radiation (kWh.m^–2^).

***(A) Substrate***: Ecological Land Unit Value: the most represented species were found on Acidic Plutonics, consisting of 16 species, which represented 84% of the total number of mapped species. At the same time, the majority of localities of mapped species were recorded on this substrate, which represented up to 56 localities out of the total of 129 mapped localities. The second most represented geological basis / substrate was carbonate sedimentary rock on which we located 10 species (77%). There were only 3 species on Metamorphic Rock, namely *Cephalanthera damasonium*, *Anacamptis morio* and *Platanthera bifolia*. The poorest substrates per species and number of localities were Non-Carbonate Sedimentary Rock (4 species) and Non-Acidic Plutonics (1 species). *Cephalanthera damasonium*, *Cephalanthera longifolia*, *Cephalanthera rubra* and *Anacamptis morio* could be included among the species with the largest ecological amplitude (Figure 5A).

***(B) Soil Reaction***: The representation of species in terms of pH value represented an interval in the range of 4.5–5.5 pH. Up to 17 species were found on soils with such a pH, of which up to 69 localities were mapped here. On the second scale in the range of 5.5–7.2 pH, there were 10 species covering 29 localities. There was only one species on the soil with a pH range of 7.2–8.5, namely *Cephalanthera longifolia*, which also had the largest ecological amplitude within the pH value of the soil (Figure 5B). The characteristic occurrence of species depending on the soil reaction (pH) indicated the nature of acid-base reactions in soils. As a result, most species were found on acidic soils, but could also be found on neutral soils.

***(C) Terrain***: Slope: Morphometric parameters of the relief, or in our case, the slope of the relief, were divided into three intervals for a better overview of the sites of the mapped species. Fifteen species were found in the scale between 3°–12°, and this scale included 44 localities out of the total number. The number of species found in the scale 13°–22° was recorded as 13, with the number of identified localities 37. In the third scale, 23°–31°, there were only 7 species covering 19 localities. From this point of view, it can be stated that the influence of the relief and its inclination did not have a significant influence on the spread of orchids in the studied area. Orchids covered mostly all types of slope as well as altitude (Figure 5C).

***(D) Flow and Hydrogeological Productivity***: Within the hydrogeological conditions, the studied area was located in two areas of flow and hydrogeological productivity. Sixteen species were located at 54 localities in the hydrogeological unit with low flow and hydrological productivity (T < 1.10–4 m^2^.s^−1^). On a mild hydrogeological unit (T = 1.10^−4^–1.10^−3^ m^2^.s^-1^) we found 10 species in 40 localities.(Figure 5D). Soil water was one of the most important ecological factors of the habitat. The total water of the terrestrial biocycle came primarily from precipitation and secondarily from groundwater. The hydrogeology of the habitat indicated that the species were largely more associated with a low range of flow and hydrological productivity.

***(E) Average Annual Amounts of Global Radiation***: Three light scales characterize the algebraic sum of fluxes of direct and scattered radiation falling on a horizontal surface, which forms a flux of global radiation of light intensity that is optimal for plants during the growing season. Most of the mapped species were found in localities with an intensity of 1100–1150 kWh.m^–2^, including the 14 species recorded in 50 localities. In the second row, the most recorded species were in the range of 1200–1250 kWh.m^–2^, 10 species with the number of identified localities at 41. The smallest number of species was found within the scale with the intensity of solar radiation at 1150–1200 kWh.m^–2^, i.e., 5 species in 13 localities. These were mainly *Cephalanthera longifolia*, *Cephalanthera rubra* and *Anacamptis morio* (Figure 5E).

## 4. Discussion

### Biogeography and Conservation Biology

Research has shown that the distribution of orchids is mostly influenced by the geology of the area and the associated soil reaction. Of the observed species, 84% were on Acidic Plutonics, which also represented the largest number of mapped species at the most recorded sites (Figure 5A). These constituted 56 localities out of the total number of 129 mapped localities (Figure 4). Another important ecological factor was the Carbonate Sedimentary Rock, on which 77% of the mapped species occurred. *Cephalanthera damasonium, Cephalanthera longifolia, Cephalanthera rubra* and *Anacamptis morio* could be included among the species with the highest ecological amplitude within the studied area. In terms of soil pH, the largest number of species were found in 69 localities in the range of 4.5–5.5 pH.

From the point of view of nature and landscape protection, the studied area creates a unique natural complex of vegetation with the occurrence of a relatively rich diversity of *Orchidaceae*. The physical and geographical conditions of the described area create a suitable perspective to ensure optimal protection of populations of protected and endangered species. Orchid populations in the area are currently endangered by human interventions, which contribute to the decline of natural habitats. Meadow species such as *Anacamptis morio* and *Orchis purpurea* are sown extensively, whereas there is no secondary succession and expansion of overgrowths to spread invasive plant species that could significantly endanger the studied populations.

The studied populations show a high degree of vitality. There are 129 localities with an occurrence of protected and endangered orchids in the investigated area, including the unprotected species *Neottia nidusavis*.

Meadow species grow on open areas of dry, xerothermic meadows. Habitat natures mostly differ in this area. Forest species such as *Cephalanthera damasonium, Cephalanthera longifolia, Cephalanthera rubra* and the genus *Epipactis* are affected by subject to active forestry activities, which have an indirect negative impact on these populations. Agriculture intesification, forestry, land drainage and soil compaction are generally factors that are significantly involved in the decline of these species. Taxa of the family *Orchidaceae* are widespread mainly throughout Europe. Their occurrence is similarly conditioned by strict protection in the surrounding states. The summarized results for the selection of European native species of vascular plants [40] indicate that the vast majority of orchids belong to Least Concern (LC), Near Threatened (NT), Vulnerable (VU) and Endangered (EN).

Within the phytogeographical division, the territory of Europe is located in the Holarctic region, the largest area in the world. It is a remnant of Tertiary flora affected by Pleistocene climate change. Within Europe, we compared the species composition [41] for countries located near Slovakia. Despite different geographical disparities, the species were almost identical in each territory. In Austria, there were *Dactytorhiza majalis, Dactytorhiza fuchsii, Orchis masculata, Platanthera bifolia, Epipactis atrorubens, Epipactis helleborine* and *Gymnadenia conopsea*. In the Czech Republic and Poland, typical representatives of the *Orchidaceae* were *Platanthera chlorantha, Platanthera bifolia, Epipactis atrotubens, Epipactis helleborine, Dactytorhiza fuchsia* and *Cephalanthera damasonium*. In Romania, there were *Gymnadenia conopsea, Platanthera bifolia, Cephalanthera longifolia* and *Anacamptis morio*. Species such as *Gymnadenia conopsea, Cephalanthera damasonium* and *Orchis mascula* were widespread in Germany. The closest studied area was in Hungary, where similar species of orchids *Orchis purpurea, Cephalanthera damasonium, Cephalanthera longifolia, Epipactis atrorubens, Anacamptis morio* and *Neottia nidus-avis* were typical for both Hungary and Slovakia. A total of 78 species have been identified in Slovakia and 70 in Hungary [42].

Furthermore, remote Turkey is a bridge between the Mediterranean lowlands and the Iran-Turan region thanks to its diverse flora. Orchids are an important part of this diversity with 191 taxa, of which 39 taxa are Turkish endemics [43].

The analysis of the localities in Table 1 and in the charts showed that the geology of the area and the associated soil reactions had the greatest influence on the distribution of orchids. 84% of the species observed were on acidic plutonics, which also represented the most recorded localities of the mapped species. These consisted of 56 localities out of the total number of 129 mapped localities. Another important ecological factor was carbonate sedimentary rock, on which 77% of the mapped species occurred (Figure 5A). *Cephalanthera damasonium*, *Cephalanthera longifolia, Cephalanthera rubra* and *Anacamptis morio* could be included among the species with the highest ecological amplitude within the studied area. In terms of soil pH, the largest numbers of the species were found in 69 localities (17/19 species) in the range of 4.5–5.5 pH. On the second scale, in the range of 5.5–7.2 pH, there were 10/19 species, covering 29 localities (Figure 5B). Only one species of *Cephalanthera longifolia* was found on soil with a pH range of 7.2–8.5. Within the morphometric parameters of the relief, 15/19 species were located in the scale between 3°–12° (44 localities). 13/19 species were found in the range of 13°–22° (37 localities). In the third scale, 23°–31°, there were only 7 species, covering 19 localities (Figure 5C).

Within the hydrogeological conditions, 16/19 species were found in 54 localities with low flow and hydrological productivity (T < 1.10^−4^ m^2^.s^−1^). Of the hydrogeological unit “mild” (T = 1.10^−4^–1.10^−3^ m^2^.s^−1^), 10 species were found within 40 mapped localities (Figure 5D). The area falls into three scales falling on a horizontal surface, which forms a flux of global radiation of light intensity that is optimal for plants during the growing season. Most of the mapped species were located in localities with an intensity of direct and scattered radiation of 1,100–1,150 kWh.m^–2^, while there were 14/19 species recorded in 50 localities (Figure 5E). The average number of species was recorded in the scale of 1,200–1,250 kWh.m^–2^ (10/19 species, with the number of identified localities 41). The smallest number of species were found in the range of 1,150–1,200 kWh.m^–2^, 5/19 species in 13 localities. These were mainly species of *Cephalanthera longifolia*, *Cephalanthera rubra* and *Anacamptis morio*. The authors [6], who carried out similar research in the Western Carpathians (Cerová vrchovina Mountains), the closest to our territory, confirmed that calcium geological substrates were an important factor, which in their case were important driving forces of orchid species diversity. They confirmed the results of previous studies, which identified carbonates and their soils as the most important substrates for the occurrence of terrestrial orchids [14,44,45]. Within our study area, a high diversity of orchids was recorded in the southwestern part, where there was a high proportion of dolomites, crystalline and corneal limestones and shales with increased calcium content. Some species were also concentrated on granodiorites and granites. Based on the soil reaction, we can say that these are types of acidic to slightly acidic soils.

Research in Ukraine [46] presents the results of the local population of *Anacamptis morio*. The size of this population is estimated at about 250–300 thousand individuals. The average density of individuals per 1 m² is 12. According to this study, *Anacamptis morio* inhabits fresh habitats of forest meadows. Its populations are found in fresh eutrophic and moist mesotrophic meadows and in coastal floodplain forests.

The American author [47] states that regional geological factors play an important role in the specialization of orchids and their biogeography. It also cites examples from northwestern South America of how evolutionary change can occur over relatively short periods of time, perhaps even as short as decades, centuries or millennia in the distribution of orchids.

Terrestrial orchids in southern Brazil [48] form a taxonomically and ecologically diverse group from tropical to subpolar areas and from moist or marshy to dry sand dunes. The occurrence of native terrestrial orchid species has been recorded for six major habitats or vegetation types: swamps and marshes, peat forests, rainforests, dune forests, baptismal stands and coastal sand dunes. The ecological range was defined for 39 species belonging to 23 genera on the basis of literature, revisions of herbariums and extensive collection along the studied area. Multivariate analyses identified light (herbaceous versus woody vegetation) as the primary ecological factor and soil drainage (sandy versus peat substrates) as the secondary factor controlling the distribution of terrestrial orchids. Despite the diametrically different continentality, we can note from the following studies that in each area, environmental factors played an important role in the distribution of orchids; further study is needed at the local level in order to predict their distribution on a global scale.

Based on the results obtained through reconnaissance (comparison of map data and terrain), we propose to regularly monitor the identified localities through changes in population ecology and analyze the results through tabular synthesis of phytocenological records, wherein there is data on species composition and quantitative shares of species at localities, as well as analysis through the so-called plant trait databases.

## 5. Conclusions

Orchid species *(Orchidaceae)* are very sensitive to environmental changes. Many species are extinct, and many are critically endangered. There are many direct reasons for their retreat; however, indirect causes have a much larger share in their disappearance from nature. The aim of our study was to map the phytodiversity of the studied area on a large scale, and further, to draw attention to the negative influences that threaten orchid populations and the abundance of individual species and then evaluate the results based on ecological environmental factors. The results of our study showed that the main ecological factors of the environment included the geological subsoil, which significantly affected the diversity of orchids.

Most of the studied orchids occur in acidophilic, calcific to basophilic habitats, i.e., they are acid-based (in soil profiles slightly to moderately supplied with water). In relation to soil acidity, it can be determined than 80% of the mapped species belong to acidophytes (pH 4.5–5.5) or mesophytes (pH 5.5–7.2).

Some species with a wide ecological amplitude grow on limestone soils—calcifytes to basifytes. The studied orchids represent hemisciophytes to sciophytes, which are mainly bound to moderately warm habitats in lower positions.

From the location of the distribution of individual populations, it can be seen that they are arranged mostly compactly, or sometimes dispersed within the country, which results in a diversity of ecological environmental factors.

In the researched area, we found that the intensification of agriculture and forestry could be classified among the negative effects on plant communities and orchid populations in the affected area. During the research, we identified 129 localities with the occurrence of 19 species and subspecies of orchids. As part of the research, we mapped 19 species of orchids occurring in the study area. Populations were recorded at 107 localities (excluding *Neottia nidusavis*, an unprotected species with a wide ecological amplitude). Despite the hypothesis of a predominance of sites on carbonate rocks, we found that this part covered only 29% of the total number of sites and 71% covered alkaline types of igneous rocks. Most localities had *Cephalanthera longifolia* 19%, *Cephalanthera damasonium* 13% and *Epipactis microphylla* 11% while the next was *Epipactis helleborine*, *Platanthera bifolia* and *Anacamptis morio* 8% and at least 1% *Gymnadenia conopsea*, G*ymnadenia odoratissima* and *Epipactis leptochila*.

Based on our findings, we recommend that these species, in the studied localities with a high abundance and diversity, in accordance with the valid Slovak legislation 356/2019 Coll., and Act no. 543/2002 Coll. on nature and landscape protection, be listed as a small protected area, resp. as a territory of European importance.

## Figures and Tables

**Table 1:** Statistical processing of ecological environment factors. *We do not analyze *Neottia nidusavis* in terms of the high number of sites and low ecological demands.

Taxon	Localities Code	Abiotic Environmental Factors				
		Substrate: Ecological Land Unit Value	Soil Reaction (pH)	Terrain: Slope (°)	Flow and Hydrogeological Productivity (m^2^.s^−1^)	Average Annual Amounts of Global Radiation (kWh.m^–2^)
***Cephalanthera damasonium***	CD_01	Carbonate Sedimentary Rock	5.5–7.2	3–12	mild (T = 1.10^−4^–1.10^-3^)	1100–1150
	CD_02	Carbonate Sedimentary Rock	5.5–7.2	13–22	mild (T = 1.10^-4^–1.10^-3^)	1100–1150
	CD_03	Carbonate Sedimentary Rock	5.5–7.2	3–12	mild (T = 1.10^-4^–1.10^-3^)	1100–1150
	CD_04	Metamorphic Rock	4.5–5.5	13–22	mild (T = 1.10^-4^–1.10^-3^)	1100–1150
	CD_05	Metamorphic Rock	4.5–5.5	13–22	mild (T = 1.10^-4^–1.10^-3^)	1100–1150
	CD_06	Acidic Plutonics	4.5–5.5	13–22	low (T < 1.10^-4^)	1200–1250
	CD_07	Acidic Plutonics	4.5–5.5	23–31	low (T < 1.10^-4^)	1200–1250
	CD_08	Acidic Plutonics	4.5–5.5	23–31	low (T < 1.10^-4^)	1200–1250
	CD_09	Acidic Plutonics	4.5–5.5	3–12	low (T < 1.10^-4^)	1200–1250
	CD_10	Metamorphic Rock	4.5–5.5	13–22	mild (T = 1.10^-4^–1.10^-3^)	1100–1150
	CD_11	Acidic Plutonics	4.5–5.5	23–31	low (T < 1.10^-4^)	1200–1250
	CD_12	Acidic Plutonics	4.5–5.5	13–22	low (T < 1.10^-4^)	1200–1250
	CD_13	Acidic Plutonics	4.5–5.5	13–22	low (T < 1.10^-4^)	1200–1250
***Cephalanthera longifolia***	CL_01	Carbonate Sedimentary Rock	5.5–7.2	23–31	mild (T = 1.10^-4^–1.10^-3^)	1100–1150
	CL_02	Carbonate Sedimentary Rock	5.5–7.2	3–12	mild (T = 1.10^-4^–1.10^-3^)	1100–1150
	CL_03	Carbonate Sedimentary Rock	5.5–7.2	13–22	mild (T = 1.10^-4^–1.10^-3^)	1100–1150
	CL_04	Carbonate Sedimentary Rock	5.5–7.2	3–12	mild (T = 1.10^-4^–1.10^-3^)	1100–1150
	CL_05	Carbonate Sedimentary Rock	5.5–7.2	3–12	mild (T = 1.10^-4^–1.10^-3^)	1100–1150
	CL_06	Acidic Plutonics	4.5–5.5	3–12	low (T < 1.10^-4^)	1200–1250
	CL_07	Non-Carbonate Sedimentary Rock	4.5–5.5	3–12	mild (T = 1.10^-4^–1.10^-3^)	1100–1150
	CL_08	Acidic Plutonics	4.5–5.5	13–22	low (T < 1.10^-4^)	1200–1250
	CL_09	Acidic Plutonics	4.5–5.5	3–12	low (T < 1.10^-4^)	1200–1250
	CL_10	Acidic Plutonics	4.5–5.5	13–22	low (T < 1.10^-4^)	1200–1250
	CL_11	Acidic Plutonics	4.5–5.5	3–12	low (T < 1.10^-4^)	1200–1250
	CL_12	Acidic Plutonics	4.5–5.5	13–22	low (T < 1.10^-4^)	1200–1250
	CL_13	Non-Acidic Plutonics	7.2–8.5	13–22	low (T < 1.10^-4^)	1200–1250
	CL_14	Acidic Plutonics	4.5–5.5	13–22	low (T < 1.10^-4^)	1200–1250
	CL_15	Acidic Plutonics	4.5–5.5	23–31	low (T < 1.10^-4^)	1200–1250
	CL_16	Acidic Plutonics	4.5–5.5	13–22	low (T < 1.10^-4^)	1200–1250
	CL_17	Acidic Plutonics	4.5–5.5	3–12	low (T < 1.10^-4^)	1200–1250
	CL_18	Acidic Plutonics	4.5–5.5	13–22	low (T < 1.10^-4^)	1150–1200
	CL_19	Acidic Plutonics	4.5–5.5	3–12	low (T < 1.10^-4^)	1200–1250
***Cephalanthera rubra***	CR_01	Carbonate Sedimentary Rock	5.5–7.2	3–12	mild (T = 1.10^-4^–1.10^-3^)	1100–1150
	CR_02	Carbonate Sedimentary Rock	5.5–7.2	23–31	mild (T = 1.10^-4^–1.10^-3^)	1100–1150
	CR_03	Carbonate Sedimentary Rock	5.5–7.2	23–31	mild (T = 1.10^-4^–1.10^-3^)	1100–1150
	CR_04	Metamorphic Rock	4.5–5.5	3–12	mild (T = 1.10^-4^–1.10^-3^)	1100–1150
	CR_05	Acidic Plutonics	4.5–5.5	23–31	low (T < 1.10^-4^)	1200–1250
***Anacamptis morio***	AM_01	Carbonate Sedimentary Rock	5.5–7.2	13–22	mild (T = 1.10^-4^–1.10^-3^)	1100–1150
	AM_02	Carbonate Sedimentary Rock	5.5–7.2	3–12	mild (T = 1.10^-4^–1.10^-3^)	1100–1150
	AM_03	Carbonate Sedimentary Rock	5.5–7.2	3 - 12	mild (T = 1.10^-4^–1.10^-3^)	1100–1150
	AM_04	Carbonate Sedimentary Rock	5.5–7.2	13–22	mild (T = 1.10^-4^–1.10^-3^)	1100–1150
	AM_05	Acidic Plutonics	4.5–5.5	3–12	low (T < 1.10^-4^)	1200–1250
	AM_06	Metamorphic Rock	4.5–5.5	3–12	mild (T = 1.10^-4^–1.10^-3^)	1100–1150
	AM_07	Acidic Plutonics	4.5–5.5	3–12	low (T < 1.10^-4^)	1200–1250
	AM_08	Acidic Plutonics	4.5–5.5	3–12	low (T < 1.10^-4^)	1200–1250
***Dactylorhiza majalis***	DR_01	Acidic Plutonics	4.5–5.5	3–12	low (T < 1.10^-4^)	1150–1200
	DR_02	Acidic Plutonics	4.5–5.5	13–22	low (T < 1.10^-4^)	1150–1200
***Dactylorhiza sambucina***	DS_01	Acidic Plutonics	4.5–5.5	3–12	low (T < 1.10^-4^)	1150–1200
***Orchis purpurea***	OP_01	Carbonate Sedimentary Rock	5.5–7.2	3–12	mild (T = 1.10^-4^–1.10^-3^)	1100–1150
	OP_02	Carbonate Sedimentary Rock	5.5–7.2	13–22	mild (T = 1.10^-4^–1.10^-3^)	1100–1150
	OP_03	Carbonate Sedimentary Rock	5.5–7.2	3–12	mild (T = 1.10^-4^–1.10^-3^)	1100–1150
***Dactylorhiza*** ***fuchsii***	DF_01	Acidic Plutonics	4.5–5.5	3–12	low (T < 1.10^-4^)	1200–1250
***Platanthera bifolia***	PB_01	Carbonate Sedimentary Rock	5.5–7.2	3–12	mild (T = 1.10^-4^–1.10^-3^)	1100–1150
	PB_02	Carbonate Sedimentary Rock	5.5–7.2	3–12	mild (T = 1.10^-4^–1.10^-3^)	1100–1150
	PB_03	Carbonate Sedimentary Rock	5.5–7.2	3–12	mild (T = 1.10^-4^–1.10^-3^)	1100–1150
	PB_04	Non-Carbonate Sedimentary Rock	4.5–5.5	3–12	mild (T = 1.10^-4^–1.10^-3^)	1100–1150
	PB_05	Metamorphic Rock	4.5–5.5	13–22	mild (T = 1.10^-4^–1.10^-3^)	1100–1150
	PB_06	Acidic Plutonics	4.5–5.5	3–12	low (T < 1.10^-4^)	1200–1250
	PB_07	Acidic Plutonics	4.5–5.5	3–12	low (T < 1.10^-4^)	1200–1250
	PB_08	Acidic Plutonics	4.5–5.5	13–22	low (T < 1.10^-4^)	1200–1250
***Platanthera chlorantha***	PCH_01	Carbonate Sedimentary Rock	5.5–7.2	23–31	mild (T = 1.10^-4^–1.10^-3^)	1100–1150
	PCH_02	Non-Carbonate Sedimentary Rock	4.5–5.5	13–22	mild (T = 1.10^-4^–1.10^-3^)	1100–1150
	PCH_03	Acidic Plutonics	4.5–5.5	3–12	low (T < 1.10^-4^)	1200–1250
***Epipactis helleborine***	EH_01	Carbonate Sedimentary Rock	5.5–7.2	23–31	mild (T = 1.10^-4^–1.10^-3^)	1100–1150
	EH_02	Carbonate Sedimentary Rock	5.5–7.2	3–12	mild (T = 1.10^-4^–1.10^-3^)	1100–1150
	EH_03	Carbonate Sedimentary Rock	5.5–7.2	13–22	mild (T = 1.10^-4^–1.10^-3^)	1100–1150
	EH_04	Acidic Plutonics	4.5–5.5	23–31	low (T < 1.10^-4^)	1200–1250
	EH_05	Acidic Plutonics	4.5–5.5	3–12	low (T < 1.10^-4^)	1200–1250
	EH_06	Acidic Plutonics	4.5–5.5	23–31	low (T < 1.10^-4^)	1200–1250
	EH_07	Acidic Plutonics	4.5–5.5	23–31	low (T < 1.10^-4^)	1200–1250
	EH_08	Acidic Plutonics	4.5–5.5	23–31	low (T < 1.10^-4^)	1200–1250
***Epipactis atrorubens***	EA_01	Carbonate Sedimentary Rock	5.5–7.2	13–22	mild (T = 1.10^-4^–1.10^-3^)	1100–1150
***Epipactis microphylla***	EM_01	Carbonate Sedimentary Rock	5.5–7.2	13–22	mild (T = 1.10^-4^–1.10^-3^)	1100–1150
	EM_02	Carbonate Sedimentary Rock	5.5–7.2	23–31	mild (T = 1.10^-4^–1.10^-3^)	1100–1150
	EM_03	Carbonate Sedimentary Rock	5.5–7.2	3–12	mild (T = 1.10^-4^–1.10^-3^)	1100–1150
	EM_04	Carbonate Sedimentary Rock	5.5–7.2	13–22	mild (T = 1.10^-4^–1.10^-3^)	1100–1150
	EM_05	Carbonate Sedimentary Rock	5.5–7.2	13–22	mild (T = 1.10^-4^–1.10^-3^)	1100–1150
	EM_06	Acidic Plutonics	4.5–5.5	3–12	low (T < 1.10^-4^)	1200–1250
	EM_07	Acidic Plutonics	4.5–5.5	3–12	low (T < 1.10^-4^)	1200–1250
	EM_08	Acidic Plutonics	4.5–5.5	3–12	low (T < 1.10^-4^)	1200–1250
	EM_09	Acidic Plutonics	4.5–5.5	13–22	low (T < 1.10^-4^)	1200–1250
	EM_10	Acidic Plutonics	4.5–5.5	23–31	low (T < 1.10^-4^)	1200–1250
	EM_11	Acidic Plutonics	4.5–5.5	13–22	low (T < 1.10^-4^)	1200–1250
***Epipactis muelleri***	EMu_01	Acidic Plutonics	4.5–5.5	13–22	low (T < 1.10^-4^)	1200–1250
	EMu_01	Acidic Plutonics	4.5–5.5	3–12	low (T < 1.10^-4^)	1200–1250
	EMu_03	Acidic Plutonics	4.5–5.5	23–31	low (T < 1.10^-4^)	1200–1250
	EMu_04	Acidic Plutonics	4.5–5.5	3–12	low (T < 1.10^-4^)	1200–1250
	EMu_05	Acidic Plutonics	4.5–5.5	13–22	low (T < 1.10^-4^)	1200–1250
	EMu_06	Acidic Plutonics	4.5–5.5	3–12	low (T < 1.10^-4^)	1200–1250
***Epipactis leptochila***	EL_01	Acidic Plutonics	4.5–5.5	13–22	low (T < 1.10^-4^)	1200–1250
***Limodorum abortivum***	LA_01	Acidic Plutonics	4.5–5.5	13–22	low (T < 1.10-4)	1200–1250
	LA_02	Acidic Plutonics	4.5–5.5	13–22	low (T < 1.10-4)	1200–1250
	LA_03	Acidic Plutonics	4.5–5.5	13–22	low (T < 1.10^-4^)	1200–1250
	LA_04	Acidic Plutonics	4.5–5.5	13–22	low (T < 1.10^-4^)	1200–1250
	LA_05	Acidic Plutonics	4.5–5.5	13–22	low (T < 1.10^-4^)	1200–1250
***Gymnadenia conopsea***	GC_01	Acidic Plutonics	4.5–5.5	3–12	low (T < 1.10^-4^)	1200–1250
***Gymnadenia odoratissima***	GO_01	Acidic Plutonics	4.5–5.5	3–12	low (T < 1.10^-4^)	1200–1250

## Data Availability

Data is contained within the article.
